# Assessing the performance of centralized waiting lists for patients without a regular family physician using clinical-administrative data

**DOI:** 10.1186/s12875-016-0573-1

**Published:** 2017-01-05

**Authors:** Mylaine Breton, Mélanie Ann Smithman, Astrid Brousselle, Christine Loignon, Nassera Touati, Carl-Ardy Dubois, Kareen Nour, Antoine Boivin, Djamal Berbiche, Danièle Roberge

**Affiliations:** 1 Charles-LeMoyne Hospital Research Centre, Sherbrooke University, Longueuil Campus, 150 Place Charles-LeMoyne, Office 200, Longueuil, QC J4K 0A8 Canada; 2 École nationale d’administration publique (Montréal), 4750, avenue Henri-Julien, Office 5117, Montreal, QC H2T 3E5 Canada; 3 Faculty of Nursing, University of Montreal, 2375, chemin de la Côte Ste-Catherine, Office 5103, Montreal, QC H3T 1A8 Canada; 4 Direction de santé publique, Centre intégré de santé et des services sociaux–Montérégie-Centre, 1255 rue Beauregard, Longueuil, QC J4K 2M3 Canada; 5 University of Montreal Hospital Research Centre, University of Montreal, 900 Rue Saint-Denis, Montreal, QC H2X 0A9 Canada

**Keywords:** Unattached patients, Centralized waiting lists, Performance, Balanced Scorecard

## Abstract

**Background:**

With 4.6 million patients who do not have a regular family physician, Canada performs poorly compared to other OECD countries in terms of attachment to a family physician. To address this issue, several provinces have implemented centralized waiting lists to coordinate supply and demand for attachment to a family physician. Although significant resources are invested in these centralized waiting lists, no studies have measured their performance. In this article, we present a performance assessment of centralized waiting lists for unattached patients implemented in Quebec, Canada.

**Methods:**

We based our approach on the Balanced Scorecard method. A committee of decision-makers, managers, healthcare professionals, and researchers selected five indicators for the performance assessment of centralized waiting lists, including both process and outcome indicators. We analyzed and compared clinical-administrative data from 86 centralized waiting lists (GACOs) located in 14 regions in Quebec, from April 1, 2013, to March 31, 2014.

**Results:**

During the study period, although over 150,000 patients were attached to a family physician, new requests resulted in a 30% median increase in patients on waiting lists. An inverse correlation of average strength was found between the rates of patients attached to a family physician and the proportion of vulnerable patients attached to a family physician meaning that as more patients became attached to an FP through GACOs, the proportion of vulnerable patients became smaller (*r* = −0.31, *p* < 0.005). The results showed very large performance variations both among GACOs of different regions and among those of a same region for all performance indicators.

**Conclusions:**

Centralized waiting lists for unattached patients in Quebec seem to be achieving their twofold objective of attaching patients to a family physician and giving priority to vulnerable patients. However, the demand for attachment seems to exceed the supply and there appears to be a tension between giving priority to vulnerable patients and attaching of a large number of patients. Results also showed heterogeneity in the performance of centralized waiting lists across Quebec. Finally, our findings suggest it is critical that similar mechanisms should use available data to identify the best strategies for reducing variations and improving performance.

**Electronic supplementary material:**

The online version of this article (doi:10.1186/s12875-016-0573-1) contains supplementary material, which is available to authorized users.

## Background

Unattached patients are patients who do not have a regular family physician (FP). These patients often rely on walk-in clinics and emergency departments to access primary care [1, 2]. Certain types of patients at higher risk of encountering barriers when accessing primary care, such as youth, recent immigrants, and patients with low incomes, little education, and/or low social support, are more likely to be unattached [3, 4]. Several studies have reported the benefits of patients being attached to an FP, such as lower emergency department use [5–7], better care coordination [8–10], greater continuity of care [11, 12], more preventive care [13, 14], better chronic disease management [15, 16], and improved health outcomes [17, 18].

With 4.6 million unattached patients, or 15.5% of its population [19], Canada performs poorly compared to other OECD countries such as Australia (12%), New Zealand (11%), Germany (8%), and the Netherlands (0%) in terms of having a regular doctor [20]. In Quebec, this issue is even more widespread, with over 25% of the population declaring not having a regular FP [19]. This issue is especially problematic given how central FPs are to the delivery of primary care and to referrals to specialist care in Canada. To address this issue, several Canadian provinces have implemented centralized waiting lists to coordinate supply and demand for attachment to an FP. These waiting lists are used to centralize requests for FPs in a given territory and match patients with physicians according to urgency of medical need and availability of primary care workforce [1, 21].

Significant resources are invested in centralized waiting lists for patients without a regular doctor across Canada. To our knowledge, no studies have measured the performance of these waiting lists, to determine whether they produce expected outcomes, or have assessed the processes that led to these outcomes. Yet it is widely recognized that measuring both outcomes and processes is essential not only to ensure accountability of such healthcare programs, but also to pinpoint areas for improvement [22, 23]. Performance assessment of centralized waiting lists is therefore essential to improve their effectiveness. In this article, we present a performance assessment of centralized waiting lists for unattached patients in Quebec, one of the first Canadian provinces to implement such mechanisms and the province with largest number of unattached patients. This article also illustrates how performance indicators can be used to monitor outcomes and processes of such mechanisms and to inform decision-makers on areas for improvement.

### Objective

The objective of this study was to assess the performance of centralized waiting lists for unattached patients implemented in Quebec, Canada, in terms of both processes and outcomes.

### Study context: Quebec’s healthcare system

Quebec’s tax-based system provides universal health insurance coverage for medical services to its eight million residents. Its healthcare system is structured on three pillars of governance. At the provincial level, the Ministry of Health and Social Services (MSSS) determines the priorities and orientations for the overall health system and ensures that the regional agencies apply the established policies. The 16 regional health and social services agencies (ASSS) are responsible for organizing and coordinating services in their respective regions. At the local level, there are 93 health and social services centres (CSSS) made up of local community services centres, long-term care facilities, and, in 85% of cases, hospitals [24]. Each CSSS is mandated to improve the health and well-being of a specific geographically-defined population [25]. In collaboration with local partners such as medical clinics, municipalities, and schools, these CSSSs ensure the delivery of services in their territory.

In Quebec, the majority of FPs work on a fee-for-service basis. Private practices are managed by FPs who are self-employed but paid by government through public health insurance. There are also local community services centres, which are government-managed and where physicians are paid on a salary basis; however, less than 20% of Quebec’s physicians work in these centres [26]. Formal attachment is a process by which patients are registered on an FP’s list of patients [27]. This facilitates accountability by defining the population for which the FP is responsible and fosters an ongoing relationship between the patient and FP [28]. Formal attachment of patients to FPs is relatively new in Quebec. It started in the early 2000s with the implementation of a new model of primary care, the Family Medicine Group model, but has since been extended to all practice settings [26, 29]. In Quebec, attachment is formalized through a written agreement which is signed by both the patient and FP. This formalization of attachment was part of a provincial government policy to add capitation-based financial payments for FPs to fee-for-service payments. Since the introduction of this new policy, most FPs have formally attached their regular patients. As of March 2014, 65% of the province’s population was formally attached to an FP [30].

### Centralized waiting lists for unattached patients in Quebec

To address the need to attach patients to FPs, in 2008 the MSSS and the Quebec Federation of General Practitioners (FMOQ) decided jointly to implement centralized waiting lists for unattached patients. The aim of these lists, called *guichets d’accès pour la clientèle orpheline* (GACO), is to facilitate access to an FP for the population of each local territory, based on a clinical priority scale and physicians’ availability to take on new patients. While the MSSS and FMOQ published a general framework to guide GACO implementation [31], in practice, there is considerable variation in GACO organization models and processes, as each of the 93 CSSSs were mandated to implement a GACO in their territory.

**Fig. 1:**
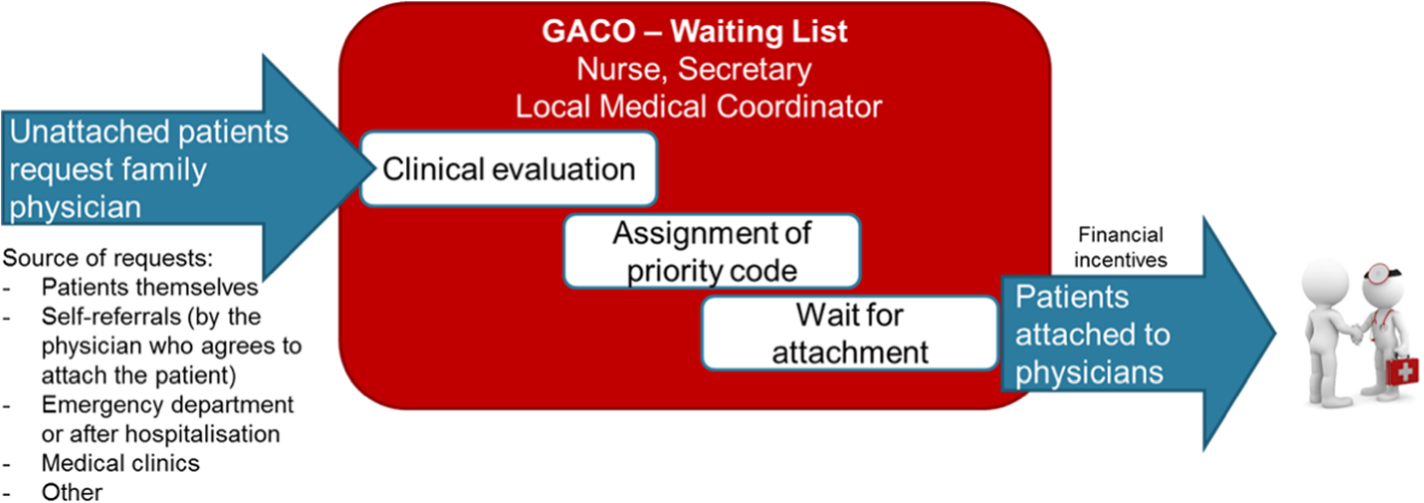
GACO Process

Figure 1 presents how GACOs generally work. Each CSSS has a local GACO. This local GACO serves the population, clinics, and FPs of the local territory. Each region has as many GACOs as it has CSSSs. In most cases, a local GACO is staffed by a secretary and a nurse working in collaboration with a medical coordinator, who is an FP from the local territory fulfilling this role in addition to his or her medical practice. Requests for an FP can be made to the GACO in several ways. Patients can make the request themselves either by phone, online, or by completing a form at a local community services centre and submitting it there or by mail. The request can also be made by a health or social services professional after seeing an unattached patient at a medical clinic or emergency department, after hospitalization, or in another care context. Finally, physicians formerly could make a request to the GACO on behalf of a patient they had agreed to attach, a process known as self-referral that was, however, later prohibited by new rules put in place by the government as of June 2013. The questions on the GACO enrolment form vary from one CSSS to another, but generally cover contact information, demographic characteristics, presence of certain medical conditions (e.g. diabetes, mental illness, hypertension), and information on health services use (e.g. emergency department visits, home care).

After the request for an FP is made to the GACO, a nurse assesses the patient’s health by phone. A priority code is then assigned to the patient by the nurse, sometimes in collaboration with the local medical coordinator, depending on the urgency and complexity of the case. Priority codes were implemented to ensure patients with the most pressing medical needs are attached to an FP first. The MSSS/FMOQ framework recommends maximum wait times for attachment to an FP for each priority level [31]. For *priority 1* cases, which require immediate medical care (complex pathologies, high risk of decompensating), the recommended wait time is 30 days or less. *Priority 2* patients should be attached within 3 months, and *priority 3* within 6 months. Patients identified as *priority 4* do not require urgent care, but should be attached to an FP within a year. For those identified as *priority 5*, considered in good health, there is no recommended wait time. Ultimately, patients are matched with an FP based on primary care workforce availability, scope of practice of FPs participating in the GACO, medical priority category, and date of request. Relevant information on the patient’s health is documented and sent to the receiving FP.

FPs’ participation in GACOs is voluntary. Those practising in the CSSS’ territory are not obligated to take unattached patients through the GACO. FPs who want to participate can contact the GACO intermittently to take on the desired number of new patients and can refuse any patient the GACO sends them. To increase the number of patients attached to an FP via GACOs and to encourage FPs to take on patients considered medically vulnerable, various financial incentives were implemented. Patients considered medically vulnerable are those who have at least one of the 20 vulnerability codes as defined by the *Régie de l’Assurance Maladie du Quebec* (RAMQ), Quebec’s health insurance board. These include 19 specific health problems (e.g. cancer, depressive disorders, intellectual disability) or being 70 years old or over [32]. Since the implementation of GACOs, financial incentives for the formal attachment of new patients have been modified on several occasions [26]. As of November 2011, FPs receive a bonus when they take on a patient via GACOs: 100$CAD for non-vulnerable patients and 200$CAD for vulnerable patients [33]. These bonuses are paid to the physician after the patient’s first visit.

## Methods

### Study design: GACO performance over one year

In this article, we assess the performance of centralized waiting lists for unattached patients (GACOs) in Quebec over a 1-year period from April 1, 2013, to March 31, 2014. We focused on this period because: a) it represented the most recent data available; b) after 5 years of implementation, GACOs could be considered firmly established in Quebec; and c) changes made to the GACO framework in previous years had major impacts on pre-2013 data [26].

### Data source: administrative databases

We analyzed clinical-administrative data from the information system related to GACOs, the *système d’information des guichets d’accès pour la clientèle orpheline* (SIGACO). This database compiles the data for all patients registered in the GACOs of the province, with the exception of two regions. Each GACO’s nurse and secretary enter the data systematically into the SIGACO database. The data used in this study were aggregated and anonymized at a local scale. We also used clinical-administrative data collected by the RAMQ to document the proportion of the general population attached to a family physician by territory, which was important contextual information for our study.

### Participants: all GACOs in the SIGACO database

In total, the data from 86 GACOs located in 14 regions of Quebec were analyzed. The seven other GACOs were excluded because their data were either unavailable or incomplete in SIGACO. The data used therefore included all patients in the SIGACO database for 86 GACOs.

### Performance indicators

**Fig. 2:**
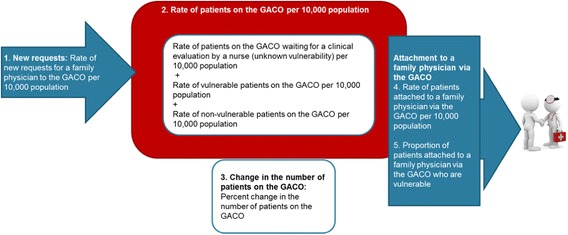
Indicators selected to assess GACOs’ performance

A committee of six decision-makers from the three levels of governance (provincial, regional and local), four healthcare professionals (two nurses and two physicians) involved in implementing and monitoring GACOs in Quebec, and four researchers from our team selected five performance indicators among those available in the SIGACO database (see Fig. 2). To identify the performance indicators presented in this article, the committee based its decision on the data from the previous year (2012–2013) and on the indicators’ usefulness in pinpointing areas for improvement of the performance of the centralized waiting lists. The committee aimed to select a limited set of indicators that comprehensively represented the GACOs’ different components (requests, prioritization, the waitlist, and attachment). Although factors external to the GACOs could have influenced the performance of several indicators, the committee selected indicators for which actions could be taken from within the GACOs to improve performance. We did not use data related to wait times for FP attachment, i.e., number of days elapsed between making a request to the GACO and becoming attached to an FP, because several irregularities were observed in the data and there were variations between regions in the way dates were recorded in the SIGACO database. Three of the selected indicators are related to the GACOs’ internal process (new requests for an FP, patients waiting for an FP, change in the number of patients waiting for an FP), while other two relate to GACO outcomes (patients attached to an FP through GACOs, vulnerable patients attached to an FP through GACOs).

### Assessing performance: use of a Balanced Scorecard

To take into account the five indicators selected by the committee, which included both process and outcome indicators, we based our approach to assessing GACO performance on the Balanced Scorecard method [23]. The initial objective of the widely adopted Balanced Scorecard was to present managers with a concise yet comprehensive overview of performance [34]. Its underlying principles are that no single measure can provide enough information to gain an understanding of critical areas of performance and that processes driving performance in achieving key outcomes must also be measured. Use of a Balanced Scorecard has been shown to improve performance by: 1) better translating strategy into operational terms; 2) transforming strategizing into a continuous process; and 3) more closely aligning an organization’s processes, services, competencies, and units [35]. The more traditional Balanced Scorecard used in the private sector, with its emphasis on financial results, may not be well suited to evaluate the performance of healthcare interventions such as centralized waiting lists. However, many organizations have adapted the Balanced Scorecard to their contexts, integrating more appropriate process and outcome measures for use in the healthcare sector [36–39]. This method allowed us, when describing GACOs’ performance, to take into account the various aspects of their processes as well as their results in terms of patient attachment to FPs and prioritization of vulnerable patients.

- ***Process indicators***
  Three process indicators were selected to describe mechanisms that precede achievement of the GACOs’ expected outcomes.
  1. *New requests for an FP*: This indicator includes every new request made in 2013–2014. It reflects the population’s and health professionals’ knowledge of the GACOs’ existence, as well as how easy it is to make a request to the GACO. The number of new requests for an FP is presented per 10,000 population. Larger numbers of new requests were considered to indicate better GACO performance.
  2. *Patients waiting for an FP in GACOs*: This rate indicates the number of pending requests for an FP as of March 31, 2014, per 10,000 population. There are three types of patients waiting for an FP in GACOs: a) patients not yet evaluated by the GACO nurse (unknown vulnerability); b) vulnerable patients, evaluated by the nurse and with at least one of the 20 vulnerability codes defined by the RAMQ (chronic disease or age 70+); and c) patients evaluated by the nurse and not vulnerable (none of the 20 vulnerability codes). We considered that, for all three types of patients, a small number indicates better GACO performance because ideally patients should be evaluated quickly by a nurse and then attached to an FP as soon as possible.
  3. *Change in the number of patients waiting for an FP in GACOs:* The number of patients on the waiting list (indicator 2) at a given date also reflects a GACO’s performance in previous years. Patients on the GACO can accumulate from year to year when there are more requests than attachments to physicians. Therefore we developed an indicator to reflect the change in number of patients on the list during 2013–2014, i.e., to capture whether the number increased or decreased over that time. This indicator is presented in both median percentage and rate per 10,000 population. This indicator captures more precisely the GACO’s performance in 2013–2014. We considered that a greater decrease in number of patients on the list reflected better GACO performance, since it indicated that more patients were attached to an FP than there were new requests for an FP during the year.
- ***Outcome indicators***
  The outcome indicators presented below reflect the dual objectives of GACOs: to attach patients to an FP and to prioritize the attachment of vulnerable patients.
  4) *Patients attached to an FP through GACOs:* This indicator measures the number of patients who were attached to an FP through GACOs during the year 2013–2014 per 10,000 population. We considered that larger numbers of patients being attached to an FP during the year indicated better GACO performance.
  5) *Vulnerable patients attached to an FP through GACOs:* This indicator measures the proportion, in percentage, of patients attached to an FP through GACOs during the year who were identified as vulnerable (with at least one of the 20 vulnerability codes). GACOs that had attached a larger proportion of vulnerable patients were considered as showing better performance, in terms of success in prioritizing those identified as having specific health needs.

#### Statistical analysis

The data were analyzed using SAS Version 9.3 software. Indicators 1 (New requests for FPs in GACOs), 2 (Patients waiting for an FP in the GACOs) and 4 (Patients attached to an FP through GACOs) were transformed into rates per 10,000 population to enable inter-region comparisons. Indicators 3 (Change in number of patients waiting for an FP in GACOs) is presented per 10,000 population and as percentages. Indicator 5 (Vulnerable patients attached to an FP through GACOs) is presented as proportions. The data are presented at the regional level. There were between 1 and 12 GACOs in each region. Descriptive statistics (central and dispersion tendencies) were calculated for each region for all indicators. Because the data did not follow a normal distribution, we present medians rather than means. We conducted multiple comparisons to test the significance of the differences between the rates per 10,000 population in each region for all five indicators. Regions were classified into tertiles of relative performance: weak (1), average (2), or strong (3), based on mean results, to see whether certain performance profiles emerged. Finally, Pearson correlation coefficients were calculated, using mean results from the lowest level of aggregated data available (i.e. local GACOs, *n* = 86), to see whether there was any correlation between the selected performance indicators. We also calculated correlation between the performance indicators and the proportion of the general population attached to an FP by local territory—an important contextual element, since it could influence supply and demand for attachment to an FP.

## Results

**Fig. 3:**
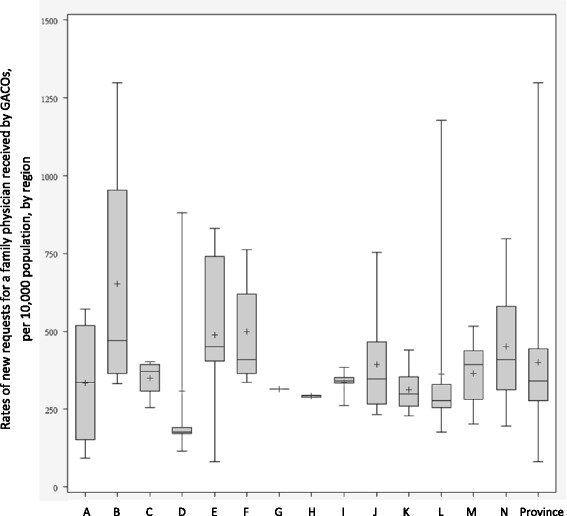
Rates of new requests for a family physician in a GACO per 10,000 population

**Fig. 4:**
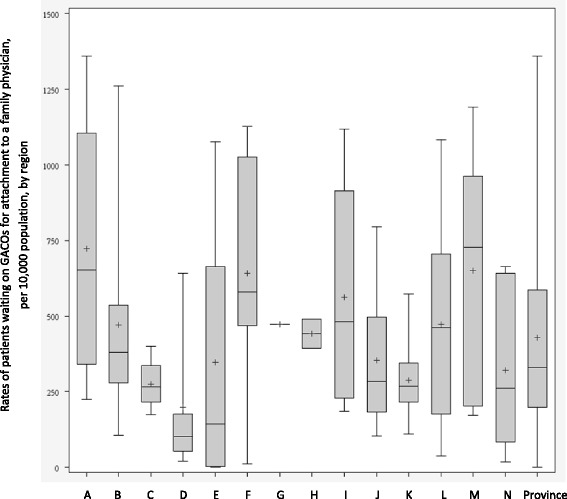
Rates of patients waiting for attachment to a family physician in the GACOs per 10,000 population

**Fig. 5:**
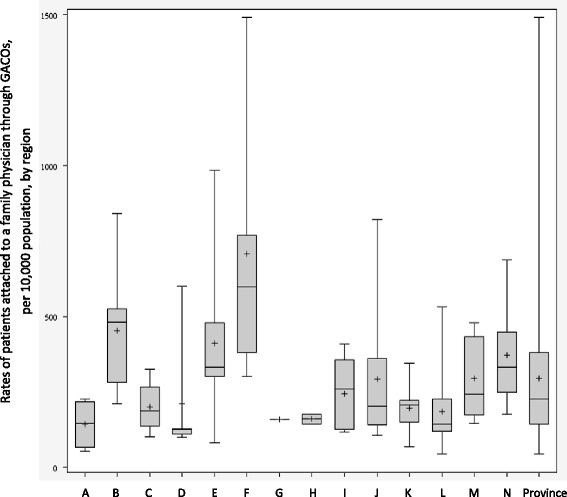
Rates of patients attached to a family physician through GACOs per 10,000 population

**Fig. 6:**
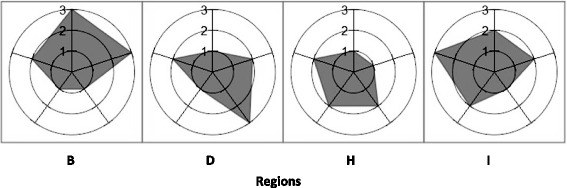
Relative performance for GACOs of four contrasting regions. The numbers indicate tertiles of relative performance: weak (1), average (2), strong (3). Position 0:00 Rates of new requests for a family physician in a GACO per 10,000 population. Position 2:24 Rates of patients attached to a family physician through GACOs per 10,000 population. Position 4:48 Rates of patients waiting for attachment to a family physician in the GACOs per 10,000 population. Position 7:12 Change in the rates of patients waiting for attachment to a family physician per 10,000 population. Position 9:36 Proportion of patients attached to a family physician through GACOs who were vulnerable

The results for each indicator are presented separately: 1) new requests for an FP through GACOs (Fig. 3); 2) patients waiting for an FP in GACOs (Fig. 4; Table 1); 3) change in number of patients waiting for an FP in GACOs (Table 2); 4) patients attached to an FP through GACOs (Fig. 5); and 5) vulnerable patients attached to an FP through GACOs (Table 3). Figures 3, 4 and 5 present the results in the form of boxplot diagrams for each region and for the province. Finally, a spider web diagram (Fig. 6) presents a synthesis of the performance of a few contrasting regions.

**Table 1:** Rates of patients waiting for attachment to a family physician in the GACOs per 10,000 population, by type

	Median rates of patients who have not been evaluated by the nurse (unknown vulnerability)	Intra-regional range	Median rates of patients who are vulnerable (at least one of 20 chronic disease vulnerability codes or aged 70+ years)	Intra-regional range
A	54	0; 208	192	56; 333
B	13	0; 696	69	24; 266
C	14	1; 36	83	73; 116
D	1	0; 149	26	7; 274
E	6	0; 193	2	0; 284
F	8	0; 128	98	1; 399
G	54	N/A	95	N/A
H	33	0; 66	45	1; 88
I	4	0; 539	81	51; 309
J	12	0; 386	80	21; 149
K	22	4; 209	27	15; 69
L	41	0; 207	137	11; 529
M	12	1; 287	122	40; 266
N	16	0; 173	45	7; 361
Province	21	0; 696	68	0; 529

**Table 2:** Change in number of patients waiting for attachment to a family physician in the GACOs

	Number of GACOs in the region	Increase/decrease in the rate of patients on the GACOs per 10, 000 population	Median change for the GACOs in the region (%)	Standard deviation (%)	Intra-regional range (best performance; worst performance) (%)
A	5	192	+22	19	+15; +56
B	8	−10	+45	38	−48; +64
C	4	185	+57	24	+19; +71
D	5	52	+46	25	+44; +91
E	7	118	+13	302	−703; +90
F	5	−188	−1	108	−231 +6
G	1	155	+33	N/A	N/A
H	2	132	+30	2	+28; +31
I	7	81	+25	22	−29;+35
J	8	147	+34	39	−67; +49
K	12	92	+41	16	+20; +65
L	11	135	+40	21	+14; +84
M	5	151	+16	41	−74; +26
N	6	79	+7	165	−364 +79
Province	86	115	+30	101	−703; +91

**Table 3:** Proportion of patients attached to an FP through GACOs who were vulnerable (%)

	Median for the GACOs in the region (%)	Intra-regional range (worst performance; best performance;) (%)
A	57	21; 68
B	48	7; 51
C	46	41; 51
D	50	24; 53
E	15	0; 46
F	46	30; 48
G	44	N/A
H	39	38;41
I	46	26; 68
J	45	27; 69
K	38	26; 69
L	39	31; 52
M	28	26; 55
N	33	27; 56
Province	41	0; 69

### Performance results: process

#### New requests for FPs in GACOs

Figure 3 represents new requests for an FP made to GACOs between April 1, 2013, and March 31, 2014, per 10,000 population. *Provincial performance:* The provincial median was 341 new requests per 10,000 population, for a total of 260,046 new requests made during the year. Most of these requests came from patients themselves (48%), a large proportion came from self-referrals (29%), and smaller numbers of requests came from health and social services centers (8%), medical clinics (6%), emergency department/hospitals (2%), and other sources (8%). *Regional performance*: The number of new requests for an FP varied widely between regions. Regional medians varied from 177 to 471 new requests per 10,000 population. Multiple comparisons showed that the differences between regions were significant. *Local performance*: During the year, one GACO received only 81 new requests per 10,000 population, in contrast to another that received nearly 1300 new requests per 10,000 population. A large variability was also observed within regions, with intra-regional ranges (between GACOs of a same region) reaching 1000 new requests per 10,000 population.

#### Patients waiting for an FP in the GACOs

Figure 4 presents cross-sectional data for the GACOs in the 14 regions on March 31, 2014. The number of patients waiting for an FP in GACOs at the end of the year 2013–2014 is the result of the number of new requests for an FP made to the GACOs since their implementation in 2008 minus the number of patients attached to an FP over that time.^1^ *Provincial performance*: On March 31, 2014, at the end of the study period, 313,364 patients were still waiting for an FP throughout the GACOs in our sample. This represents a median of 329 patients waiting for an FP in GACOs per 10,000 population. *Regional performance*: At the regional level, median rates of patients waiting for an FP in GACOs varied from 101 to 728 per 10,000 population, with multiple comparisons showing largely significant differences between the regions. *Local performance*: Numbers of patients waiting for an FP varied widely among GACOs. Indeed, within a same region, one GACO might have no patients waiting while another might have more than 1000 waiting per 10,000 population.

Table 1 presents the different types of patients waiting for attachment to a family physician in the GACOs per 10,000 population. The results show a large variation in the median rates of vulnerable patients waiting for an FP in GACOs, with certain regions, such as E, D, and K, displaying relatively low rates and others, such as regions A, L, and M, displaying relatively high rates. Moreover, the rate of vulnerable patients may have been underestimated in regions where there was a relatively high rate of patients not yet evaluated by a nurse, such that their vulnerability codes were not known, as in regions A and G.

#### Change in the number of patients waiting for an FP in GACOs

Table 2 presents the median, standard deviation, and range of change in numbers of patients waiting for attachment to an FP in GACOs during 2013–2014. Positive values indicate increases in patients on the list and negative values, decreases. *Provincial performance*: This indicator revealed, at the provincial level, a 30% median increase in patients waiting in the GACOs for a total of 107,421 additional patients waiting in the GACOs. *Regional performance*: There was wide inter-regional variation for this performance indicator, with region F showing a median decrease of 0.72% in patients waiting for an FP in its GACOs over the year and five other regions (B, C, D, K, L) showing median increases of more than 40%. Multiple comparisons of each region’s median rate revealed largely significant differences. *Local performance*: The size and direction of change in number of patients waiting for an FP varied widely across regions: within a same region, the GACO with the worst performance for this indicator showed an increase of over 90%, whereas the GACO with the best performance reduced its waiting list by more than 700%.

#### Patients attached to an FP through GACOs

This indicator, the rate of patients attached to an FP through GACOs in 2013–2014, is represented in Fig. 5. *Provincial performance:* During the study period, 152,625 patients were attached to an FP via the GACOs under study, with a provincial median of 226 patients per 10,000 population. *Regional performance:* Regional medians varied from 125 to 598 patients per 10,000 population. Multiple comparisons showed largely significant differences between regions. *Local performance:* Numbers of patients attached to an FP via GACOs varied considerably, ranging from 45 to 1492 patients per 10,000 population. Within a same region, the variance could be as large as 1192 patients per 10,000 population.

#### Vulnerable patients attached to an FP through GACOs

As shown in Table 3, amongst the patients attached to an FP through GACOs, the proportion of patients who are vulnerable varied widely. *Provincial performance:* Provincially, 61,229 patients attached to an FP through GACOs during the study year were identified as having at least one of the 20 vulnerabilities listed by the RAMQ, with a median 41% of vulnerable patients provincially. *Regional performance*: At the regional level, the median proportion of vulnerable patients attached to an FP through GACOs varied from 15 to 57%. Multiple comparisons showed significant differences between certain regions. *Local performance*: The proportion of patients attached to an FP through the GACO who were vulnerable varied considerably, ranging from 0 to 69%. Within a same region, in one GACO only 7% of patients attached to an FP were vulnerable, whereas that proportion was over 50% in three other GACOs.

### Overview of relative performance for all five indicators

For each of the five indicators, we classified regions into tertiles of relative performance, where tertile 1 indicated relatively weak performance and tertile 3 indicated relatively strong performance. No region was classified in the strongest or weakest performance tertiles for all five indicators. Region B was the region most often classified in the strong performance tertile (4 times out of 5). Region I ranked twice in the strongest performance tertile and three times in the average tertile. At the opposite end, region G was the region that ranked most often in the weakest (twice) and average (three times) tertiles. Region D ranked twice in the weakest tertile, twice in the average tertile, and once in the highest tertile. Figure 6 presents an overview of these five indicators for these contrasted cases. No clear performance patterns emerged from classifying the regions into performance tertiles.

### Correlations between indicators

The results for the Pearson correlation coefficients were calculated at the local GACO level (i.e. the lowest level of data aggregation, *n* = 86). As such, indicator 1 (New requests for an FP made to the GACOs) was strongly and significantly correlated with indicator 2 (Patients waiting for an FP in GACOs) (*r* = 0.51 *p* <0.0001), meaning that as requests for an FP increased, more patients were waiting. Indicator 1 was also strongly correlated to indicator 4 (Patients attached to an FP through GACOs) (*r* = 0.61, *p* < 0.0001), meaning that as new requests increased, more patients became attached to an FP through GACOs. Indicator 3 (Change in number of patients waiting for an FP in GACOs) had a strong inverse correlation with indicator 4 (*r* = −0.53, *p* < 0.0001), meaning that as more patients became attached to an FP, the number of patients waiting for an FP decreased. There was a significant inverse correlation of average strength between indicator 5 (Vulnerable patients attached to an FP through GACOs) and indicator 4 (*r* = −0.31, *p* < 0.005), meaning that as more patients became attached to an FP through GACOs, the proportion of vulnerable patients became smaller. No significant correlation was found between the five performance indicators and the proportion of each territory’s population attached to an FP.

## Discussion

### Performance of GACOs at the outcome and process levels

Like centralized waiting lists implemented in other Canadian provinces, the GACOs have the twofold objective of: 1) attaching patients to a family physician, and 2) giving priority to vulnerable patients [21]. The results presented in this article show that, in 2013–2014, the number of new requests for an FP made to GACOs throughout Quebec (260,046 requests) largely exceeded the number of patients who became attached to an FP through GACOs in that period (152,625 patients). This may mean the GACOs’ outreach was good, but that FPs’ availability or participation in GACOs (supply) was insufficient to meet the demand. Results also show that, in total, nearly 22% of patients waiting for an FP were vulnerable, whereas this proportion rose to almost 40% among patients who were attached to an FP through GACOs in 2013–2014. Vulnerable patients therefore seem to have been given priority in attachments to FPs. Overall, the GACOs seemed to have: 1) attached a large number of unattached patients to FPs even though the overall number of requests exceeded capacity, and 2) prioritized vulnerable patients in attachments to FPs.

### Heterogeneity of GACOs’ performance

The results showed very significant differences in performance between regions and large variations both among GACOs of different regions, and among those of a same region. GACOs’ performance varied with regard to indicators related to objectives (Patients attached to an FP through GACOs; Vulnerable patients attached to an FP through GACOs), as well as indicators reflecting processes (New requests for an FP made to GACOs; Patients waiting for an FP in GACOs; Change in number of patients waiting for an FP in GACOs). No region was classified in the strongest performance tertiles for all five indicators. Also, GACOs of a same region could perform relatively well for one indicator and not for others. The large variations in performance may be explained, in part, by the fact that the MSSS and FMOQ provided very few details on how the CSSS should implement the GACOs, “leaving each CSSS considerable latitude in the strategies they adopted” and leading to “large variability in what services GACOs offer” [21]. It is possible that implementing such a mechanism with few guidelines is one of the reasons we have observed such heterogeneity in the performance of GACOs.

Therefore, the results indicate there is room for improvement in GACOs’ performance for all the regions under study. Such improvement is necessary to reduce the large performance heterogeneity observed across the province—heterogeneity that can lead to important inequalities in access to an FP between regions and even between territories of the same region.

### Correlations between indicators and levers for improvement

The correlations between indicators offer clues regarding paths to explore in identifying levers for improving GACOs. The number of new requests for an FP, i.e., the influx of patients into GACOs, appears to be an important performance indicator, as it was strongly correlated with the rate of patients waiting for an FP and with the rate of patients attached to an FP through GACOs. Moreover, the negative correlation of average strength between the rate of patients attached to an FP via GACOs and the proportion of these patients who are vulnerable indicates there may exist a certain tension between the two objectives of the GACOs. Indeed, it appeared that, as the number of patients being attached to an FP grew, the proportion of these patients who were vulnerable declined. Surprisingly, the GACO performance indicators did not seem to be correlated with the proportion of unattached patients on the territory, which we expected would have a significant influence on performance.

### Limitations

Clearly, the five indicators presented in this article paint an incomplete picture of GACO processes and outcomes, the choice of indicators being limited by the data available in the clinical-administrative database used. While these indicators enabled us to obtain a quick and comparable overview of the GACOs’ performance, developing performance indicators that reflect more accurately the processes and the outcomes of GACOs could help to better identify areas of improvement. For instance, since the indicators presented in this article come from aggregated data, we did not have information on the patients’ individual profiles, such as their specific health issues (mental health, hypertension, HIV/AIDS, etc.). We cannot know if the vulnerable patients who were attached to an FP were in fact the most vulnerable ones, i.e., those with multiple health problems or with the most complex health problems. Moreover, the indicators presented here are measures of processes and of immediate outcomes only, since the database that was used only focuses on the period extending from patients’ request for an FP to their attachment. Therefore, we are unable to present medium- or long-term outcome measures of the GACOs, such as use of healthcare services after attachment to an FP, or retention of the FP. A study comparing use of healthcare services before and after attachment to an FP through GACOs could assess whether GACOs actually improve access to primary care. Finally, the process and outcome indicators used can help identify performance variations and potential areas for improvement, but are insufficient to understand the source of the variation. Qualitative case studies comparing the processes and mechanisms of GACOs with relatively strong performance against those with relatively weak performance on certain indicators would be necessary to gain a better understanding of the sources of variations both from within the GACOs and from external local or regional influences.

### Usefulness of the Balanced Scorecard

The advantage of the Balanced Scorecard approach is that it allowed us to assess and compare the performance of the GACOs by using a combination of indicators that reflect multiple aspects of processes and outcomes. If, conversely, we had used only one indicator, such as the percentage of requesting patients who were subsequently attached to an FP through GACOs, we would lose relevant process information. Focusing on the large percentage might, for instance, obscure the fact that the actual number of requests processed by the GACO was small. Using multiple indicators also allowed us to take into account the GACOs’ twofold objective (attach patients and prioritize vulnerable patients) in evaluating their performance as well as various processes that provide details on the GACOs’ mechanisms.

The Balanced Scorecard is an interesting tool to use in the context of significant performance heterogeneity. No GACO in our study ranked in the strongest performance tertile for all indicators. It is therefore impossible to verify what the “best” GACO does and to develop general recommendations for all other GACOs based on what this “best” GACO is doing. However, it is relevant for a GACO with a relatively weaker performance for one indicator, such as prioritizing vulnerable patients in attachments to FPs, to know how another GACO that has a relatively strong performance for this indicator is doing. Comparing GACOs’ performance on various indicators can thereby stimulate a reflexive practice to improve processes and outcomes.

However, the tool itself is not sufficient. It has to be combined with communal reflection on the standards to be set for each indicator: How many new requests should be targeted? What proportion of attached patients should be vulnerable? An understanding of the Balanced Scorecard content and deliberation among the actors involved in the GACOs are necessary to improve the overall performance of GACOs [35–37]. Moreover, using a combination of indicators, each with their particular strengths and weaknesses, raises the question of whether certain indicators are more important than others and whether some should be given greater weight in evaluating overall performance. Is a GACO with a large number of new requests and a large number of patients being attached to an FP, but with a small proportion of vulnerable patients in that number, performing as well as a GACO that has a small number of new requests, and hence fewer patients being attached to an FP, but of whom a large proportion are vulnerable patients? To adequately assess the performance of GACOs in Quebec, in all their complexity, it will therefore be necessary to clearly define strategic priorities and performance standards for each indicator and to adjust the Balanced Scorecard accordingly. In this way, the Balanced Scorecard can go above and beyond a simple assessment of performance and become a reflexive tool to improve GACOs’ processes and outcomes.

## Conclusion

In this article, we assessed the performance of centralized waiting lists for unattached patients in Quebec, the Canadian province with the largest proportion of unattached patients. There are a number of conclusions that other provinces or countries looking to put in place similar mechanisms to help patients find a regular family physician should take away from the Quebec experience. First, these mechanisms can be an effective way to attach a large number of patients to FPs, including vulnerable patients. However, depending on the context, they may not meet the demand for attachment which may result in large waiting lists. Second, there is a large heterogeneity in the performance of these mechanisms across Quebec, which may in part be due to the province having mandated implementation at a local level without clear guidelines. This heterogeneity may lead to unequal access to an FP for populations of different territories. Third, there may be a trade-off between attaching a large number of unattached patients and prioritizing vulnerable patients. Finally, our findings suggest it is critical that similar mechanisms should use available data to stimulate reflection on the best strategies for reducing variations and improving performance.

## Additional file

Additional file 1: GACO Performance Data. Medians and ranges for each indicator by region and for the province of Quebec; significance testing for differences between regions; Pearson correlations between indicators. (XLSX 27 kb)

## Abbreviations

ASSS: Agence de la santé et des services sociaux/Health and Social Services Agency
CSSS: Centre de santé et des services sociaux/Health and Social Services Centre
FMOQ: Fédération des médecins omnipraticiens du Québec/Quebec Federation of General Practitioners
GACO: Guichet d’accès pour la clientèle orpheline/Centralized wait list for unattached patients
MSSS: Ministère de la santé et des services sociaux/Ministry of Health and Social Services
RAMQ: Régie de l’assurance maladie du Québec/Quebec Health Insurance Board
SIGACO: Système d’information des guichets d’accès pour la clientèle orpheline/GACO Information System

## Footnotes

1 Death and relocation of patients can also reduce the waiting list. However, because our analysis was comparative, we assumed similar attrition in all GACOs.
